# Development and validation of a stability-indicating HPLC method for the simultaneous determination of anticoccidial drugs in veterinary formulations: greenness and whiteness assessment

**DOI:** 10.1038/s41598-024-84849-4

**Published:** 2025-02-12

**Authors:** Michel Y. Fares, Shimaa Ebrahim Abdel Aziz, Israa Abdelghafar khalil, Asmaa Othman El Demerdash, Neven M. Habib

**Affiliations:** 1 Department of Pharmaceutical Chemistry, Faculty of Pharmacy, Nahda University in Beni-Suef (NUB), Sharq El-Nile, Beni-Suef, 62511 Egypt; 2 Department of Analytical Chemistry, Faculty of Pharmacy (Girls), Al-Azhar University, Nasr City, 11884 Egypt; 3 Department of Pharmaceutical Analytical Chemistry, Faculty of Pharmacy, Beni-Suef University, Alshaheed Shehata Ahmed Hegazy St., Beni-Suef, 62574 Egypt

**Keywords:** Amprolium, Sulfaquinoxaline, Diaveridine, Vitamin K3, HPLC, Greenness assessment, Analytical chemistry, Drug therapy, Biological techniques, Chemical biology, Chemistry

## Abstract

Intestinal coccidiosis is a significant parasitic disease affecting poultry, resulting in substantial economic losses for the industry. It compromises the nutrition absorption, leading to weight loss and elevated mortality rates. Furthermore, the stress caused by the infection can compromise the immune system, making poultry more susceptible to secondary infections and reducing overall productivity. As a result, simple analytical techniques are critical for determining anticoccidial drugs. A new, sensitive, and environmentally friendly HPLC method was developed for determining amprolium (AMP), sulfaquinoxaline (SUL), diaveridine (DIV), and vitamin K3 (VIT K3) in their formulations for the first time. Stability tests were performed under diverse stress conditions to verify the safety and efficiency of the formulation throughout its designated shelf time. These investigations ascertain the influence of various environmental conditions on a drug’s chemical stability and physical characteristics. A Supelcosil C18 column was used as the stationary phase, and 0.05 M KH_2_PO_4_ and acetonitrile were mixed in a ratio of 80:20 (v/v) as the developing system with a flow rate of 2.0 mL min^−1^. The proposed drugs were quantified at 260 nm. It was tested and found that the novel analytical method was linear for AMP and SUL between 20.0 and 60.0 µg mL^−1^, 2.0–6.0 µg mL^−1^ for VIT K3, and 2.1–6.3 µg mL^−1^ for DIV. The anticipated method was validated according to ICH guidelines. Advanced evaluation tools, such as GAPI, Red Green Blue (RGB 12, whiteness), Blue Applicability Grade Index (BAGI), the Analytical Eco-Scale, and (AGREE) assessed the sustainability profile of the proposed method, illustrating its enhanced environmental friendliness and sustainability.

## Introduction

Amprolium-HCl (AMP) is a pyrimidine derivative that prevents carbohydrate synthesis by inhibiting thiamine metabolism. So it is used to prevent and treat intestinal coccidiosis^1^. It is commercially available in formulations for veterinary use only, alone or in combination with ethopabate, sulfonamides, or pyrimethamine^2^.

Sulfaquinoxaline-Na (SUL) stops the production of dihydrofolic acid, which is why it is often used to treat intestinal coccidiosis. Although it has a broad spectrum of activity against several types of bacteria and protozoa, resistance is common when used alone^3^.

Diaveridine-HCl (DIV) is a synthetic dihydrofolate reductase inhibitor that interferes with folic acid metabolism in different manners, resulting in remarkable activity against coccidia^4,5^. So, it works better against bacteria when mixed with sulfonamides or other germ-killing substances, which stops the growth of resistant strains^6^.

Vitamin K3 (menadione), (VIT K3), is a synthetic derivative of the naturally occurring phylloquinone (K1). It is well known that VIT K3 is required for the synthesis of prothrombin and coagulation factors to prevent hemorrhage^7^. It is also the first choice in VIT K deficiency caused by malabsorption syndrome, cholestasis, protozoal disease causing diarrhea, etc.^8^.

Intestinal coccidiosis, a severe disease in veterinary medicine, can be fatal due to its severe symptoms, including diarrhea, weight loss, and decreased poultry production. Anticoccidials or vaccinations are crucial for control and prevention. However, prolonged use has led to resistant strains. Researchers suggest anticoccidial drug combinations with different modes of action, resulting in slight cross-resistance^9^. This study focuses on the AMP, VIT K3, DIV, and SUL mixture.

The study aimed to analyze AMP, VIT K3, DIV, and SUL simultaneously using the HPLC method in their mixtures, as other analytical techniques were not reported for simultaneous determination.

The literature review showed that AMP and DIV with 24 other anticoccidial compounds were determined in water samples using the UHPLC–MS/MS method^10^. AMP and VIT K3 were simultaneously determined by the HPLC method^11^. Four approaches were used to simultaneously determine AMP and SUL. This binary mixture was determined through two HPLC procedures^12,13^. Additionally, TLC-densitometry was employed for their assay^13^. Additionally, two mathematical methods dealing with the ratio spectra of the two drugs were suggested, including the double divisor ratio derivative and the ratio dual wavelength^14^.

Micellar electrokinetic chromatography was performed for the analysis of the AMP, SUL, and DIV mixture^15^. Despite the high efficiency and precision of this technique, it may suffer from poor reproducibility due to electroosmotic flow and temperature changes^16,17^. For the spectrophotometric analysis of this ternary mixture used dual-wavelength ratio spectra, partial least-squares, and principal component regression approaches were used for their quantification^18^.

Another ternary mixture comprised of SUL, DIV, and VIT K3 was analyzed by chemometric models^19^. The applied univariate methods were successive ratio subtraction coupled with constant multiplication, absorbance subtraction, and amplitude modulation. While the developed multivariate involved principal component regression and partial least-squares. Additionally, HPLC method was published for separating the three medicines in their veterinary formulations^20^.

Simultaneous estimation of AMP, SUL, and VIT K3 by hydrophilic interaction liquid chromatography^21^ is based on one of the important approaches for separating polar molecules. However, a limitation was observed: asymmetric peak shapes and poor efficiency would be observed owing to injecting a sample solution with a higher elution strength than the mobile phase. For example, the injection solvent contains a higher ratio of water (required for dissolving polar samples), while the mobile phase contains a higher proportion of an organic solvent^22^.

The stability-indicating studies of the cited drugs were also taken into our consideration. Evidence from the literature showed three HPLC methods relying on the previously mentioned hydrophilic interactions principle. Each of AMP, SUL, and VIT K3 was effectively separated individually from their degradation products. The applied elution liquid was a mixture of ammonium acetate solution and acetonitrile in different proportions^23–25^. Although no method was documented for the analysis of DIV in the presence of its degradation products, an LC–MS method was developed for the identification of in vivo DIV metabolites in chickens in order to understand its metabolic pathway^26^.

Green analytical chemistry encourages cleaner, safer, and more effective analytical procedures, which is in line with the worldwide trend toward sustainability. It is a vital path for the future of chemical analysis, as it not only protects the environment but also improves laboratory safety, efficiency, productivity, and regulatory compliance. Hence, the presented study evaluated eco-friendliness and sustainability using different tools: Green Analytical Procedure Index (GAPI)^27^, Blue Applicability Grade Index (BAGI)^28^, whiteness assessment^29^, and Analytical GREEnness Metric tool (AGREE)^30^. Furthermore, these assessment tools were employed for all the reported methods, confirming their outstanding eco-friendliness^10–15,18–21,23–25^.

Clearly, it can be seen that the mixture of AMP, VIT K3, DIV, and SUL had never been analyzed simultaneously by any analytical technique. Furthermore, no stability studies were proposed for these quaternary components together. This work aims to improve a stability-indicating RP-HPLC method for the simultaneous quantification of AMP, VIT K3, DIV, and SUL, as well as their stressed degradation products. Furthermore, this work aimed to assess the environmental performance and sustainability of the method through the use of greenness, blueness, and whiteness metrics.

## Experimental

### Instrumentation

Analysis was accomplished using Agilent 1200 series HPLC (Agilent Technologies, CA, USA). This instrument was kitted with an auto-sampler (G1367C), a quaternary pump (G1312A), a column temperature regulator (G1316A) and a UV detector (G1315B).

### Chemicals and reagents

AMP and SUL were provided by Hangzhou Longshine Bio-Tech CO., Ltd. (China) with purity of 98.17% and 99.2%, respectively. Also, VIT K3 and DIV were provided by Across Biotech Jinan Co., Ltd. (China) and Nanhai Beisha Pharmaceutical Co., Ltd. (China) with purity of 99.1% and 98.39%, respectively. The mentioned purity for the four drugs was according to the certificate provided by supplier.

Treatcox® produced and supplied by Arab company for gelatin and pharmaceutical products (**Arab-Caps**), labelled to contain (20 gm AMP, 20 gm SUL, 2.0 gm VIT K3 and 2.1 gm DIV per 100 gm powder).

Acetonitrile (HPLC grade) was acquired from Sigma Aldrich (Steinheim, Germany) and potassium dihydrogen phosphate was bought from Merck (Germany).

### Chromatographic conditions

HPLC analysis of the four proposed drugs was conducted on Supelcosil-C18 column with the following characters (4.6-mm × 25-cm, 5 μ) using isocratic elution. The used mobile phase was a 0.05 M KH_2_PO_4_: Acetonitrile (80:20, v/v) mixture with a flow rate of 2.0 mL min^−1^. At ambient temperature, 10 µL of the solution was injected into the column three times. The UV detection was achieved at a wavelength of 260 nm, Table S1.

### Standard solutions

The preparation of the stock standard solution involved the transfer of 500 mg AMP, 50 mg VIT K3, 52.5 mg DIV, and 500 mg SUL into a 25 mL volumetric flask, which was then dissolved in the chosen diluent (90% acetonitrile in water) and completed to the mark with the same diluent.

Working standard solutions of 200 µg mL^−1^ AMP, 20 µg mL^−1^ VIT K3, 21 µg mL^−1^ DIV, and 200 µg mL^−1^ SUL were prepared in 100 mL volumetric flasks by adding one mL of stock solution and the volume was filled to the mark using the diluent.

### Linearity and construction of calibration curve

To investigate the linearity range of each drug, different serial dilutions of working standard solutions were made in the range of 20.0–60.0 µg mL^−1^ of AMP and SUL, 2.0–6.0 µg mL^−1^ of VIT K3 and 2.1–6.3 µg mL^−1^ of DIV (i.e. 50–150% of test concentration) then injected into the HPLC system in triplicate, as stated in "Chromatographic conditions".

A calibration curve was constructed by plotting peak area values against the four proposed drug concentrations and then calculating the regression equation. In accordance with the ICH guidelines, the analytical method was validated for linearity, accuracy, precision, limit of quantitation, limit of detection, specificity, and robustness.

### Stress stability studies

These studies were performed under different acidic, basic, oxidative, photolytic, and thermal conditions according to ICH guidelines^31^.

#### Acid hydrolysis

AMP, VIT K3, DIV, and SUL standards weighing 200 mg, 20 mg, 21 mg, and 200 mg were added to a 100 mL volumetric flask, followed by the addition of 5 mL of 1 N HCl, and heated on a water bath at 80 °C for one hour, then pH adjusted to 7.0 with 1 N sodium hydroxide. Complete dissolving was achieved by adding 70 mL of diluent followed by sonication for 5 min, then diluted to volume using the same diluent. In a 50 mL volumetric flask, one mL of the previous solution was added and completed to the mark using the diluent.

#### Base hydrolysis

The procedure described above in acid hydrolysis was performed substituting HCl with NaOH and neutralizing with HCl instead of NaOH.

#### Oxidative hydrolysis

AMP, VIT K3, DIV, and SUL standards weighing 200 mg, 20 mg, 21 mg and 200 mg were separately mixed with 0.5% H_2_O_2_ followed by heating in water bath at 80 °C for 60 min. The resulting solution was evaporated completely then dissolved in 70 mL of diluent followed by sonication for 5 min, then diluted to volume using the same diluent. In a 50-mL volumetric flask, one mL of the previous solution was added and completed to the mark using the diluent.

#### Photodegradation

Photo-stability was assessed by separately subjecting the standard drug solutions; AMP (40 µg mL^−1^), VIT K3 (4 µg mL^−1^), DIV (4.2 µg mL^−1^), and, SUL (40 µg mL^−1^) to 48 h of direct sunlight and 12 h of UV radiation, followed by filtration through a syringe filter.

#### Thermal degradation

It was studied by heating the drug solutions (at the same concentrations used in photo-stability study) in a water bath at 80 °C for 8 h, allowing them to cool, and then filtering them through a syringe filter.

### Assay of pharmaceutical preparations

One gram of the pharmaceutical dosage form (Treatcox® powder) was dissolved in 70 mL of diluent in a 100 mL volumetric flask and the mixture was sonicated for 5 min to dissolve and then completed to the mark. One mL of the solution was transferred into a 50 mL volumetric flask, diluted to volume, mixed using a diluent, and filtered using 0.45 µm pore size filter paper. The final concentration will be AMP (40 µg mL^−1^), VIT K3 (4 µg mL^−1^), DIV (4.2 µg mL^−1^), and SUL (40 µg mL^−1^).

## Result and discussion

The authorization and registration of new pharmaceutical formulations primarily required the development of validated assay techniques accompanied by comprehensive stability and specificity studies to anticipate the recently generated degradation products and the physicochemical parameters influencing the stability of the studied drugs and formulations^32^.

The following manuscript presented a novel validated and stability-indicating HPLC approach for the assessment of AMP, VIT K3, DIV, and SUL simultaneously. Moreover, the study was accompanied by an exhaustive assessment into the greenness, blueness, and whiteness profiles of the analytical method in comparison with other reported approaches. According to the literature review, neither reported analytical techniques nor stability studies were proposed for the simultaneous evaluation of such quaternary components.

### Method development and optimization

Following several trials, adequate and optimal chromatographic separation of the cited compounds from their forced degradation products and other related substances was achieved in order to achieve the best resolution and well-defined symmetry of all eluted peaks within the appropriate total runtime.

Various combinations of the mobile phase were experimented with; primarily, ethanol, methanol, and acetonitrile were employed as organic solvents. Unfortunately, broad and overlapped peaks with poor separation from the degradation products were observed with both ethanol and methanol. However, peak shape, resolution factor, and retention time were much improved when acetonitrile was tried, resulting in enhanced separation of the eluted substances. Accordingly, acetonitrile was applied as the organic phase. When potassium dihydrogen phosphate was used as an aqueous part of the mobile phase instead of sodium heptane sulfonate, noise or drifting could not be seen, and the baselines were more stable.

The low viscosity of the mobile phase did not necessitate elevation of column temperature; additionally, better outcomes were obtained at ambient temperature (approximately 28 °C). The flow rate was fixed at 2.0 mL min^−1^. It is worth mentioning that the usage of 90% acetonitrile in water as a diluent critically improves the chromatographic performance and assures the stability of prepared solutions along the proposed chromatographic assay.

Two analytical columns were tested as stationary phases; Kinetex-C18 (2.6 μm, 4.6 mm × 100 mm, 100 A) and SUPELCOSIL-C18 (4.6-mm × 25-cm, 5 μ). The last one showed a significantly improved resolution. The optimal detection wavelength for the cited compounds was detected following scanning at several wavelengths (220, 254, 260, and 280 nm) in terms of sensitivity. Satisfactory response and higher sensitivity with minimum noise for all the proposed analytes were attained at 260 nm.

**Fig. 1:**
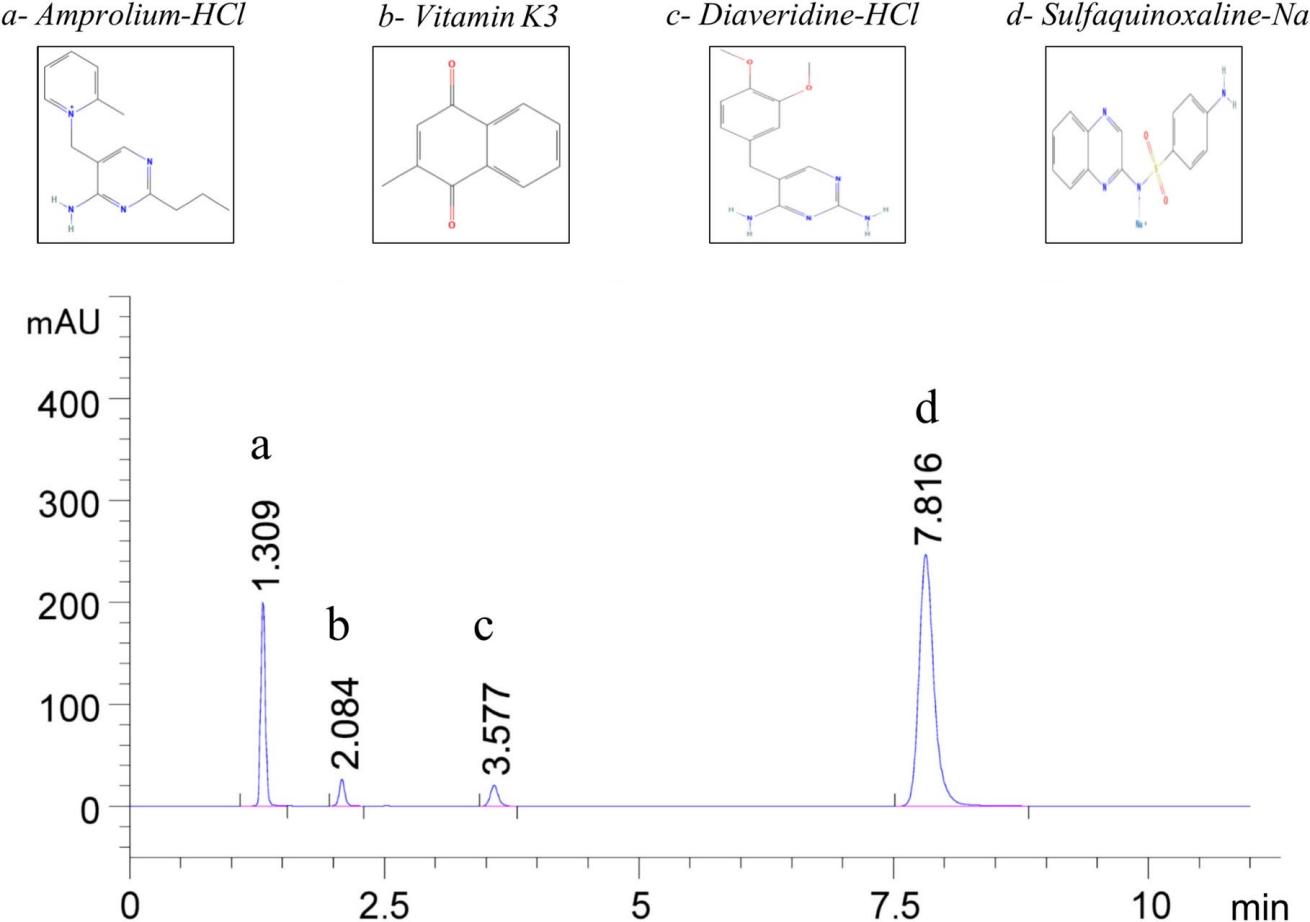
Chromatogram for separation of a standard mixture of (**a**) AMP 40 µg mL^−1^, (**b**) VIT K3 40 µg mL^−1^, (**c**) DIV 4.2 µg mL^−1^, and (**d**) SUL 40 µg mL^−1^.

The optimal chromatographic separation of the four compounds and their degradation products was achieved under standard chromatographic conditions as illustrated in Table S1. Figure 1 showed a typical chromatogram of the four drugs; AMP, VIT K3, DIV and SUL at retention times 1.309 ± 0.011, 2.084 ± 0.008, 3.577 ± 0.021 and 7.816 ± 0.056 min, respectively.

System suitability parameters including selectivity, peak symmetry, column efficiency, and resolution were computed^32^ for the proposed HPLC method as given in Table S2.

### Degradation behavior of the studied components

**Fig. 2:**
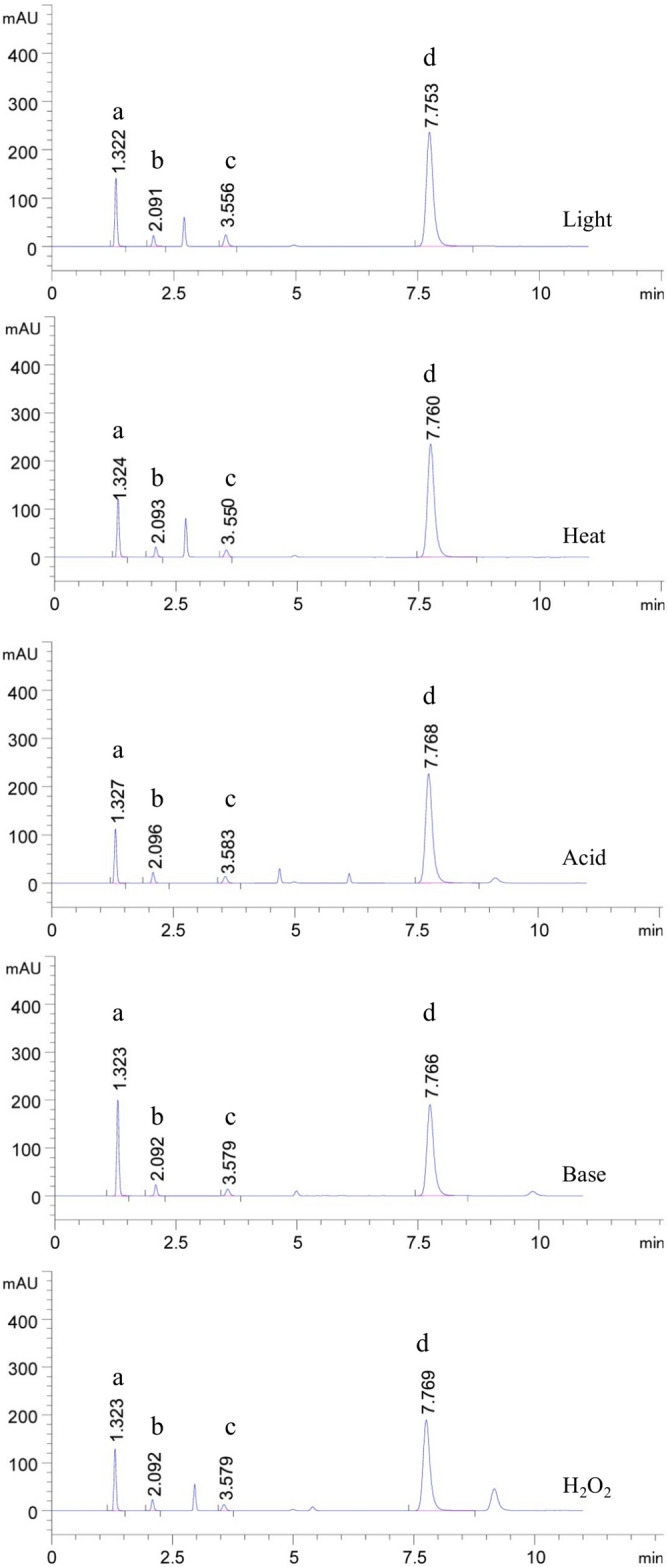
HPLC chromatograms of AMP, VIT K3, DIV, and SUL after different stress conditions: (1) photolytic degradation, (2) thermal degradation, (3) acidic degradation, (4) alkaline degradation, and (5) oxidative degradation.

The proposed chromatographic method was effectively applied to recognize the stability of the four drug standards upon exposure to several forced degradation conditions including; photodegradation, acidic and basic hydrolysis, oxidative degradation, and thermal degradation where the peaks of the cited drugs were successfully resolved from the generated degradation products as showed in the chromatograms presented in Fig. 2.

AMP was stable against acidic and basic hydrolysis, whereas it yielded about 26.96% degradation during photodegradation. Unfortunately, maximum degradation was observed during both oxidative and thermal degradation (34.06–38.92%). VIT K3 yielded about 8% upon basic hydrolysis and a slightly higher degradation of 12.78–17.11% under other degradation conditions. Likewise, DIV was not influenced by the photodegradation, whereas acidic and basic conditions induced nearly 28.01–29.14% degradation. Thermal degradations resulted in a 24.87% decrease in the parent drug peak area. Additionally, the highest degradation 33.36% was markedly recorded under oxidative conditions. Conversely, SUL demonstrated stability against thermal degradation, acidic hydrolysis, and photolysis. However, it yielded about 19% degradation during both basic hydrolysis and oxidative degradation. It is worthwhile to mention that the degradation consequences of AMP, SUL and VIT K3 concerning the forced conditions yielded maximum degradation percentage herein were consistent with the data mentioned in some reported stability-indicating studies^23–25^.

**Fig. 3:**
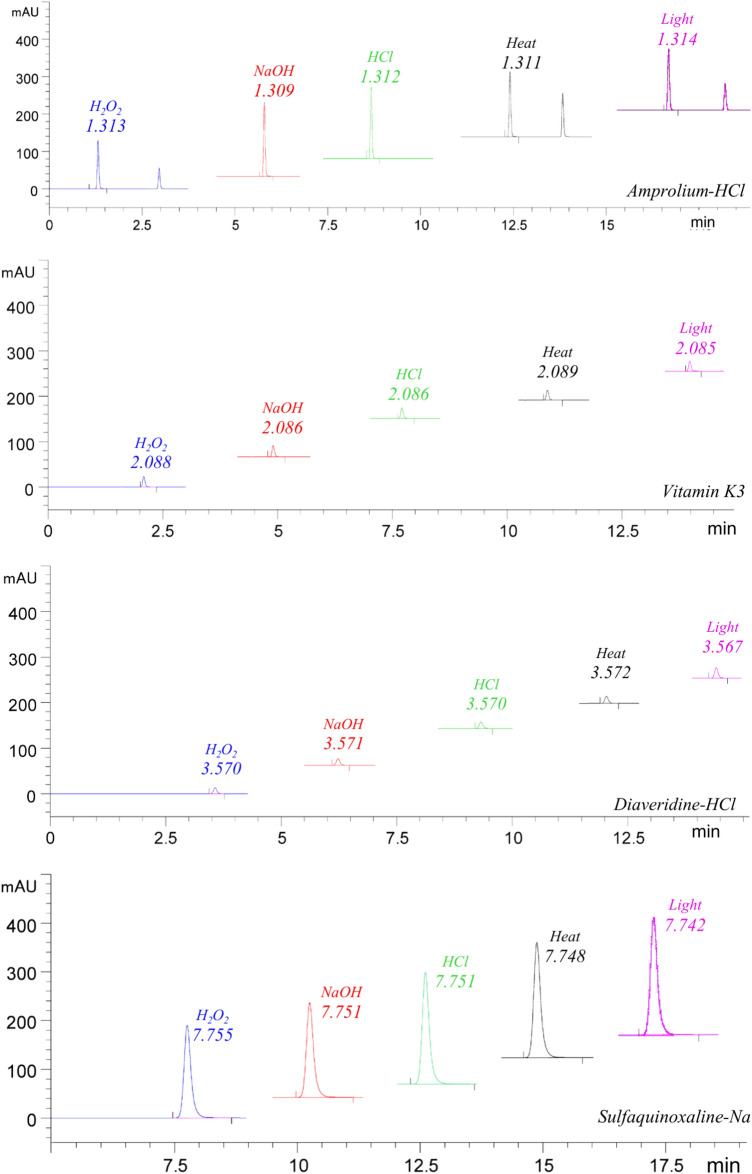
Forced degradation of AMP, VIT K3, DIV, and SUL under different stress conditions.

No interference was found at the retention time of mean peaks of AMP, VIT K3, DIV and SUL after acid, alkali, H_2_O_2_, Heat and Light treatment. The alterations in the peak of each parent analyte regarding its shape and response following the exposure to the above-stated degradation conditions were showed in Fig. 3. So the method is found to be stability indicating method for the analysis of AMP, VIT K3, DIV and SUL in WSP. The exhaustive forced degradation profile for the four compounds is presented in Table S3.

### Validation of the suggested method

The validation of the proposed method was achieved in compliance with the International Council for Harmonization (ICH) guidelines^33,34^.

The linearity of the proposed HPLC procedure was assessed by analysis of different concentration levels of the four drugs using the above-mentioned chromatographic settings detailed in Table S1. Acceptable linearity for AMP, VIT K3, DIV, and SUL was obtained within the concentration ranges of 20.00–60.00 µg mL^−1^, 2.00–6.00 µg mL^−1^, 2.10–6.30 µg mL^−1^ and 20.00–60.00 µg mL^−1^ with correlation coefficients (r) of 0.9995, 0.9998, 0.9998 and 0.9999; respectively. The range covers from 50 to 150% of the standard solution after dilution. Table 1 expresses the obtained data.

**Table 1:** The analytical method validation items for AMP, VIT K3, DIV, and SUL according to ICH guidelines^33,34^.

Validation item	AMP	VIT K3	DIV	SUL	Reference value
Range (µg mL^−1^)	20.0–60.0	2.0–6.0	2.1–6.3	20.0–60.0	–
Regression equation	y = 14.933x + 14.878	y = 23.934x + 1.007	y = 25.503x + 0.2647	y = 60.621x + 14.148	–
R	0.9995	0.9998	0.9998	0.9999	r ≥ 0.99
Precision RSD%	0.27	0.58	0.9	0.21	RSD ≤ 2%

Specificity	Well-resolved peaks from each other and degradation products				No interference
LOD (µg mL^−1^)	1.87	0.11	0.125	0.757	According to the formula "3.3xσ/S
LOQ (µg mL^−1^)	5.666	0.333	0.378	2.293	According to the formula "10xσ/S
Robustness	2.29	1.61	1.55	1.77	Combined RSD ≤ 3% in each option
Ruggedness					Combined RSD ≤ 3% in each option
Different days	1.65	1.48	2.35	2.32	
Many analysts	1.48	1.49	1.99	2.26	

The method’s accuracy was estimated using a triplicate of three concentrations; 50%, 100%, and 150% of the corresponding concentration range for each drug. It was found to range between 98.72 and 100.64%, 98.77–101.74%, 99.55–101.63% and 98.30–101.77% for AMP, VIT K3, DIV, and SUL; respectively. Acceptable recovery percentages were observed, approving the accuracy of the method, Table S4.

The obtained regression equations from the calibration curves of the four studied analytes were used to calculate LOQ and LOD based on the standard deviation of the response and the slope as presented in Table 1. The low values of LOQ and LOD ensured the sensitivity of the proposed HPLC technique and confirmed its capability for further assessment of the drug’s traces in biological and environmental matrices.

The method’s precision was performed utilizing six replicates of the identical standard solution within the corresponding linearity range of each drug. It was evaluated by calculating the RSD%, which was found to be not more than 2%, indicating the repeatability and reproducibility of the investigated methods. Table 1.

The method’s specificity was determined for AMP, VIT K3, DIV, and SUL standards, Treatcox® WSP powder, and placebo. The chromatograms show no interference. The obtained data was considered acceptable and confirmed the selectivity and specificity of the recommended method, Table S5. The specificity of the method was further verified by examining the generated degradation products; enough chromatographic separation and clearly delineated peak shapes for AMP, VIT K3, DIV, and SUL and their degradates under the above-described degradation conditions. This revealed the stability-indicating feature of the proposed method.

Ruggedness of the method was performed by the analysis of the samples under a diversity of environments. The selected variations were; different days and different analysts. Three replicates of a single concentration of each drug (40 µg mL^−1^ for AMP, 4 µg mL^−1^ for Vit K3, 4.2 µg mL^−1^ for DIV and 40 µg mL^−1^ for SUL) were executed on the first day. On the second day, three replicates of freshly prepared samples at the same concentration levels were analyzed. The same analyst performs both tests to estimate day-to-day ruggedness. Furthermore, using the abovementioned concentrations for each drug, two equal samples were prepared and analyzed by two analysts using the same conditions to evaluate analyst-to-analyst ruggedness. As shown in Table 1, the resulting pooled RSD of all drugs was not more than 3% confirming the ruggedness of the developed method.

Robustness of the method was accomplished by the analysis of the samples under minor intended changes in the approved chromatographic parameters. Two parameters were examined: condition 1 was concerned with a minor change in mobile phase composition (± 2%). Condition 2 included minor modifications in the pH of the aqueous phase (4 ± 0.02) where the same analyst performs both tests. No significant effects on the peak areas of the four compounds were observed. The results presented in Table 1 showed that pooled RSD did not exceed 3%. Hence, the proposed method can be considered robust.

System suitability parameters of the proposed chromatographic settings were computed. As summarized in Table S2. The results were convenient with a satisfactory range stated in their corresponding reference values^35^ confirming the optimal selectivity and total baseline separation of the investigated chromatographic system.

### Application to veterinary preparations

The devised HPLC method was effectively applied for the determination of Treatcox® WSP powder comprising the four cited drugs without interference from the labeled inactive ingredients, namely, polyethylene glycol as solubilizer, anhydrous sodium carbonate as buffering solution, and lactose monohydrate as formulation carrier. No co-eluted inactive substances were noticed, and the peaks of the four drugs were revealed at the expected retention times without alteration in the peak shape and resolution. The recoveries were 102.45%, 100.06%, 100.95%, and 101. 70 for AMP, VIT K3, DIV, and SUL, respectively.

Clearly, the developed HPLC method is appropriate for the estimation of the target drugs in their commercial formulation, requiring minimal sample preparations with good recovery outcomes.

### Comparison with other developed methods

The literature survey exposed limited approaches for the concurrent quantification of tertiary mixtures of AMP, VIT K3, and SUL^19–21^ and AMP, DIV, and SUL^15,18^. Further methodologies were reported for the determination of two of our targeted drugs^10–13^, as detailed in Table 2. Based on the novel combination of AMP, VIT K3, DIV, and SUL in a single dosage form, the simultaneous determination of the cited four drugs has not been published yet. Additionally, the shorter total time of analysis of the targeted analytes offering time-saving features to our developed method surpassed other reported methods^10,11,15,20,21^.

**Table 2:** Comparative study between some of the previously published studies and the proposed study.

Target analyte	Method type	Dosage form	Range	Mobile phase & elution technique	Detection (nm)	Run time (min.)	Ref. No.
AMP DIV	UHPLC–MS/MS	Water samples	0.5–250 0.5–150 ng L^−1^	gradient elution of ammonium format with 0.01% formic acid in water (A) and 0.1% formic acid in acetonitrile (B)	MS/MS	12	^10^
AMP VIT K3	HPLC	Oral solutions	1.25–5.00 0.01–0.03 mg mL^−1^	Sodium heptane sulfonate: methanol: acetonitrile (50: 45:5; v/v/v)	PDA at 254	45	^11^
AMP SUL	HPLC	Powders	0.08–0.12 mg mL^−1^	Phosphate buffer: acetonitrile (30:70; v/v)	UV at 263	7	^12^
	UHPLC	Powders	0.5–25.0 1.0–30.0 μg mL^−1^	Sodium hexane sulfonate: methanol: acetonitrile (15:4:1, v/v/v), pH 5.1	UV at 263	10	^13^
	TLC		1.0–30.0 1.0–25.0 μg/band	CH_3_Cl: methanol: 33% ammonia (6:4:0.5; v/v/v)	UV at 263	–	
	Double divisor ratio derivative & Ratio dual wavelength	Powders	6.0–50.0 3.0–25.0 μg mL^−1^	Solvent: Methanol	UV	–	^14^
AMP SUL DIV	Micellar electrokinetic chromatography	Powders	1.0–20.0 0.5–25.0 μg mL^−1^	Tris buffer (pH 8.5) with SDS: acetonitrile (85:15, v/v)	At 28 kV & 200 nm	14	^15^
	Ratio dual-wavelength & two Multivariate chemometrics	Premix	1.0–20.0 0.5–10.0 0.5–19.0 μg mL^−1^	Solvent: Methanol	UV	–	^18^
SUL DIV VIT K3	Univariate & Multivariate	Powders	1.0–10.0 1.0–11.0 1.0–8.0 μg mL^−1^	Solvent: Distilled water	UV	–	^19^
	HPLC	Powders	0.5–30 μg mL^−1^	Methanol: acetonitrile: water (20:20:60, v/v/v)	UV at 230	13	^20^
AMP SUL VIT K3	HPLC	Powders	12–26 0.12–0.26 μg mL^−1^	Ammonium acetate buffer: acetonitrile (15:85; v/v), pH 5.7	UV at 263	20	^21^
AMP VIT K3 DIV SUL	HPLC	Powders	20–60 2–6 2–6 20–60 μg mL^−1^	0.05 M KH_2_PO_4_: Acetonitrile (80:20; v/v)	UV at 260	10	Proposed study
AMP	HPLC Stability indicating	Powders	12–36 μg mL^−1^	Ammonium acetate solution: acetonitrile (25:75; v/v), pH 5.7	PDA at 267	12	^23^
SUL	HPLC Stability indicating	Powders	12.5–37.5 μg mL^−1^	Ammonium acetate solution: acetonitrile (10:90; v/v), pH 5.7	UV at 263	14	^24^
VIT K3	HPLC Stability indicating	Injectable Solutions	15–45 μg mL^−1^	Ammonium acetate solution: acetonitrile (20:80; v/v), pH 5.7	PDA at 261	12	^25^
DIV	LC–MS Metabolites	In vivo in Chicken	–	Gradient elution of 0.1% aqueous formic acid (A) and methanol (B)	MS	16	^26^

### Comprehensive evaluation of greenness, blueness, and whiteness

Given that analytical methodologies provide invaluable information regarding potential economic and environmental impacts, it is imperative to assess their eco-friendliness. But it is a broad concept that incorporates a variety of ideas, including the effectiveness of costs, safety issues, methods for minimizing waste, and ecological responsibility^36^. This study integrates complimentary evaluation methodologies to assess the eco-friendliness of the proposed method along with other reported ones using greenness, blueness, and whiteness assessments^37^.

#### Greenness assessment

By limiting or eliminating the usage of chemical hazards, green chemistry seeks to reduce the dangers that pollutants, chemicals, and/or toxins can bring to the environment and public health^31^. To assess the level of ecological sustainability attained by the proposed HPLC method, the Green Analytical Procedure Index (GAPI) was used^27^.

GAPI is a semi-quantitative tool that provides a thorough ecological evaluation of the whole analytical process considering fifteen different variables. It provides details on every step of the analytical process from sampling to the analysis final step. GAPI offers a certain symbol with five pentagrams to assess and measure the environmental effects associated with each stage of an analytical method utilizing the green, yellow, and red color codes, which represent low, medium, and high impacts on the environment, respectively.

Evaluation of the proposed HPLC method using GAPI tool showed that it had 3 green colored parts, 8 yellow, and 4 red as shown in Table 3. Hence, the method had little risk to the environment and possessed an adequate level of greenness. Referring to the other reported methods^10–15,18–21,23–25^, they were also evaluated using GAPI tool, and the obtained pentagrams were represented in Table 3. Their ecological impacts on the surrounding environment were not all the same.

**Table 3:** Results of the used green metrics for the proposed method and the selected reported methods.

Target analyte	Method Type	GAPI	Eco-Scale	Agree	Blueness	Whiteness	Ref. No.
Amprolium-HCl Diaveridine-HCl	UHPLC–MS/MS		86				^10^
Amprolium-HCl Vitamin K3	HPLC		76				^11^
Amprolium-HCl Sulfaquinoxaline-Na	a-HPLC		90				^12^
	b-UHPLC		86				^13^
	c-TLC		78				
	d-Spectrophotometry Double divisor ratio derivative and Ratio dual wavelength		81				^14^
Amprolium-HCl Sulfaquinoxaline-Na Diaveridine-HCl	a-Micellar electrokinetic chromatography		86				^15^
	b-Spectrophotometry Dual wavelength in ratio spectra		81				^18^
Sulfaquinoxaline-Na Diaveridine-HCl Vitamin K3	a-Spectrophotometry Univariate and Multivariate		87				^19^
	b-HPLC		84				^20^
Amprolium-HCl Sulfaquinoxaline-Na Vitamin K3	HPLC		84				^21^
Amprolium-HCl	HPLC Stability indicating		89				^23^
Sulfaquinoxaline-Na	HPLC Stability indicating		89				^24^
Vitamin K3	HPLC Stability indicating		89				^25^
Amprolium-HCl Sulfaquinoxaline-Na Diaveridine-HCl Vitamin K3	HPLC		86				Presented Study

#### Blueness assessment

In contrast to tools that are primarily focused on greenness, the recently developed BAGI provides a quantitative assessment of an analytical method’s “blueness” which depends on other important practical criteria^28^. In order to provide a comprehensive assessment of the blueness or suitableness of the analytical method, BAGI takes into account ten essential factors: analysis type, number of analytes, number of analyzed samples in one hour, used reagents, required instrumentation, number of simultaneously examined samples, pre-concentration steps, level of atomization, amount of analyzed sample and its preparation. BAGI assessment results in certain pictograms with value at the centre. The pictogram’s hue scale demonstrates the degree of adherence to the stated standards; dark blue indicates high compliance, blue indicates moderate compliance, light blue indicates poor compliance, and white indicates no compliance. The calculated total score of the blueness of the analytical technique is shown as a value in the centre of the BAGI pictogram, which varies from 25 to 100. The most ineffective method has a score of 25, conversely, a score of 100 indicates the method’s exceptional execution.

In our study, the HPLC method had a high score of 80, proving good practical application, high productivity, extensive possibilities for automation, and inexpensive running costs. On the other hand, BAGI scores of the previously published methods^10–15,18–21,23–25^ were also evaluated to assess their applicability as shown in Table 3. Most of them exhibited high BAGI scores revealing an elevated potential for application.

#### Whiteness assessment

White analytical chemistry is seen as a more comprehensive extension of green analytical chemistry. Additionally, greenness, it takes into account other significant criteria impacting the analytical approaches, such as analytical and practical features. It uses the RGB color model, which blends red, green, and blue light rays to give the white impression^29^. The red color in the RGB model denotes the effectiveness of the created technique, while the green one represents features of green chemistry. Whereas the blue color is associated with time and money efficiency. Red, green, and blue tables on an Excel template sheet need to be filled up with the proper scores. To assess the developed method’s whiteness, a numerical value out of 100 is computed. The resulting chart displays the percentage of each color as well as the color white, which is the consequence of combining them. To optimize the analytical method, high percentages of the three colors especially white, are very necessary.

The proposed HPLC method had elevated scores of 87.5, 87.5, and 90.8% for red, green, and blue colors, respectively. The total whiteness score was 88.6%, Table 3. Hence, the proposed method had an appropriate degree of quality and greenness. This tool was also applied to other reported methods^10–15,18–21,23–25^, where different whiteness scores were obtained as shown in Table 3 illustrating the variation in their compliance with white chemistry.

#### AGREE and eco-scale assessment

The AGREE score, a software-based analytical greenness tool that can be used quantitatively and rapidly, represents the 12 guiding codes of green analytical chemistry^30^. The more environmentally friendly the process, the greater the score achieved. Table 3 results indicate that the proposed HPLC method achieves a score of 0.64 in comparison to existing HPLC methods. The employment of poisonous and corrosive solvents in the documented processes is the primary reason for the low rating (steps 11 and 12 in AGREE metric software). The Eco-scale score^38^ is a semi-quantitative instrument that evaluates method greenness using a scoring system. The assessed parameters were primarily contingent upon the quantity of pictograms. The analytical eco-scale score is derived by subtracting the cumulative penalty points of the utilized solvents, instruments, and generated waste from 100. The new HPLC method achieved the highest eco-scale score (86), as indicated in Table 3, in comparison to previously reported methods.

### Constraints of the study and prospective research

The suggested technique was unable to identify the degradation products that were produced. Therefore, in order to obtain a complete structural explanation and a purity profile for each degradation product, it is recommended to carry out NMR and MS investigations.

Definitely, the development of a single validated analytical technique for the adequate separation and determination of such four compounds in a single run is a high economic feature regarding the cost and time of analysis. Currently, the validated HPLC method that has been developed serves as a unique and suitable method for routine analysis of AMP, VIT K3, DIV, and SUL in their respective dosage forms. Despite the stability study utilized in the current assay, it did not involve the necessary research to clarify the structure elucidation of the degradation products, however, it greatly helps in predicting the degradation profile and stability of the new combined dosage form under different stress conditions.

## Conclusion

The development of a new, sensitive, and environmentally friendly HPLC method for the determination of four anticoccidial drugs marks a significant advancement in the management of intestinal coccidiosis in poultry. Stability tests conducted under various stress conditions further validate the robustness of the formulations, ensuring their effectiveness throughout their shelf life. A comparative analysis of the greenness profile of the developed method versus previously reported methods demonstrates its low negative environmental impact. Compliance with ICH guidelines and a favorable sustainability profile, assessed through advanced evaluation tools, underscore the method’s potential for widespread application in the poultry industry. Ultimately, this innovation is poised to significantly improve poultry health, reduce economic losses, and promote sustainable practices within the sector. Future research should focus on applying this method in field studies and exploring its adaptability to other veterinary pharmaceuticals, thereby enhancing its utility and impact.

## Supplementary Information

Supplementary Information.

## Data Availability

The original data can be obtained from the corresponding author (Michel Y. Fares; Michel.yousry@nub.edu.eg) upon a reasonable request.
