# Supplementary material for: Development and validation of a stability-indicating HPLC method for the simultaneous determination of anticoccidial drugs in veterinary formulations: greenness and whiteness assessment
Source: Sci Rep. 2025 Feb 12;15:5190. doi: 10.1038/s41598-024-84849-4 (PMC11821846; doi:10.1038/s41598-024-84849-4)
Supplement: Supplementary file 1 — Supplementary Information. [file 41598_2024_84849_MOESM1_ESM.docx]

**Development and Validation of a Stability-Indicating HPLC Method for the Simultaneous Determination of Anticoccidial Drugs in Veterinary Formulations: Greenness and Whiteness Assessment**

**Shimaa Ebrahim Abdel Aziz ^a^,**

**Israa Abdelghafar khalil ^a^,**

**Asmaa Othman El Demerdash ^a^**

**Neven M. Habib ^b^**

**Michel Y. Fares ^c^**

^a^ Department of Analytical Chemistry, Faculty of Pharmacy (Girls), Al−Azhar University, Nasr City 11884, Egypt.

*b Department of Pharmaceutical Analytical Chemistry, Faculty of Pharmacy, Beni-Suef University, Alshaheed Shehata Ahmed Hegazy St., 62574 Beni-Suef, Egypt.*

*c Department of Pharmaceutical Chemistry, Faculty of Pharmacy, Nahda University in Beni-Suef (NUB), Sharq El-Nile, 62511 Beni-Suef, Egypt.*

***^⁎^Corresponding author.***

***E-mail address:*** michel.yousry@nub.edu.eg ***(Michel Y. Fares).***

**Tel:** +20-127-450-5687

**Abstract**

Intestinal coccidiosis is a significant parasitic disease affecting poultry, resulting in substantial economic losses for the industry. It compromises the nutrition absorption, leading to weight loss and elevated mortality rates. Furthermore, the stress caused by the infection can compromise the immune system, making poultry more susceptible to secondary infections and reducing overall productivity. As a result, simple analytical techniques are critical for determining anticoccidial drugs. A new, sensitive, and environmentally friendly HPLC method was developed for determining amprolium (AMP), sulfaquinoxaline (SUL), diaveridine (DIV), and vitamin K3 (VIT K3) in their formulations for the first time. Stability tests were performed under diverse stress conditions to verify the safety and efficiency of the formulation throughout its designated shelf time. These investigations ascertain the influence of various environmental conditions on a drug's chemical stability and physical characteristics. A Supelcosil C18 column was used as the stationary phase, and 0.05 M KH_2_PO_4_ and acetonitrile were mixed in a ratio of 80:20 (v/v) as the developing system with a flow rate of 2.0 mL min-1. The proposed drugs were quantified at 260 nm. It was tested and found that the novel analytical method was linear for AMP and SUL between 20.0 and 60.0 µg mL^-1^, 2.0 to 6.0 µg mL-1 for VIT K3, and 2.1 to 6.3 µg mL^-1^ for DIV. The anticipated method was validated according to ICH guidelines. Advanced evaluation tools, such as GAPI, Red Green Blue (RGB 12, whiteness), Blue Applicability Grade Index (BAGI), and (AGREE) assessed the sustainability profile of the proposed method, illustrating its enhanced environmental friendliness and sustainability.

**Keywords**

Amprolium; Sulfaquinoxaline; Diaveridine; Vitamin K3; HPLC; Greenness Assessment.

**Table S1: Chromatographic conditions for the proposed HPLC method**

| Column | SUPELCOSIL C_18_ (4.6-mm×25-cm, 5μ) |
| --- | --- |
| Mobile phase | 0.05M KH_2_PO_4_: Acetonitrile (80:20) |
| Elution Mode | Isocratic |
| Flow rate | 2.0 mL min^-1^. |
| Inj. Volume | 10.0 μL |
| Temp | Ambient |
| Detector | UV at 260 nm |

**Table S2: System suitability parameters for the proposed HPLC method**

| Parameter | AMP | VIT K3 | DIV | SUL | REF value (a) [35] |
| --- | --- | --- | --- | --- | --- |
| Selectivity (α) | - | 1.59 | 1.7 | 2.23 | ˃ 1.0 |
| Resolution (Rs ) | - | 7.97 | 11.83 | 21.12 | Rs ˃ 1.5 |
| Column efficiency (N) | 3665 | 6979 | 9439 | 14109 | The higher the more efficient the column |
| Symmetry | 0.87 | 0.87 | 0.82 | 0.75 | Not more than 2 |
| Retention time (min) | 1.309± 0.011 | 2.084± 0.008 | 3.577± 0.021 | 7.816± 0.056 | - |

**Table S3: The percentage of degradations and recoveries of** **AMP, VIT K3, DIV, and SUL following the application of stressful degradation conditions**

| Degradation Mode | Degradation Condition | AMP | | VIT K3 | | DIV | | SUL | |
| --- | --- | --- | --- | --- | --- | --- | --- | --- | --- |
|  |  | Assay % | Deg % | Assay % | Deg % | Assay% | Deg % | Assay % | Deg % |
| Light | Light (48 hrs.)/  UV (12 hrs.) | 73.04 | 26.96 | 84.58 | 15.42 | 99.88 | 0.12 | 99.45 | 0.55 |
| Heat | 80°C (8 hrs.) | 61.08 | 38.92 | 82.89 | 17.11 | 75.13 | 24.87 | 99.04 | 0.96 |
| Acid | 1 N HCl/  80°C (1 hr.) | 99.52 | 0.48 | 87.22 | 12.78 | 71.99 | 28.01 | 95.99 | 4.01 |
| Base | 1 N NaOH/  80°C (1 hr.) | 99.76 | 0.24 | 92.00 | 8.00 | 70.86 | 29.14 | 80.16 | 19.84 |
| Oxidation | 0.5% H_2_O_2_/  80C (1 hr.) | 65.94 | 34.04 | 87.68 | 12.32 | 66.64 | 33.36 | 80.93 | 19.07 |

**Table S4: Accuracy data for AMP, VIT K3, DIV, and SUL by HPLC method**

|  | AMP | | | VIT K3 | | | DIV | | | SUL | | |
| --- | --- | --- | --- | --- | --- | --- | --- | --- | --- | --- | --- | --- |
| Conc | Observed peak area | Average | %R | Observed peak area | Average | %R | Observed peak area | Average | %R | Observed peak area | Average | %R |
| 50% | 310.9 | 307.7 | 100.64% | 47.8 | 48.1 | 101.25% | 53.5 | 54.4 | 101.63% | 1226.9 | 1224 | 101.28% |
|  | 306 |  |  | 47.8 |  |  | 56.7 |  |  | 1218.4 |  |  |
|  | 306.2 |  |  | 48.8 |  |  | 53 |  |  | 1227.8 |  |  |
| 100% | 622.7 | 620.8 | 101.52% | 96.6 | 96.8 | 101.74% | 108.9 | 108.5 | 101.39% | 2468.5 | 2461 | 101.77% |
|  | 616.6 |  |  | 96.7 |  |  | 108.9 |  |  | 2463.2 |  |  |
|  | 623 |  |  | 97 |  |  | 107.9 |  |  | 2449.9 |  |  |
| 150% | 908.2 | 905.5 | 98.72% | 140.5 | 140.9 | 98.77% | 158.4 | 159.9 | 99.55% | 3568.6 | 3565 | 98.30% |
|  | 908.3 |  |  | 141.1 |  |  | 160.1 |  |  | 3562.3 |  |  |
|  | 899.8 |  |  | 141 |  |  | 161.2 |  |  | 3563.6 |  |  |

**Table S5: Specificity data for AMP, VIT K3, DIV, and SUL by HPLC method**

| AMP | | | VIT K3 | | | DIV | | | | SUL | | |
| --- | --- | --- | --- | --- | --- | --- | --- | --- | --- | --- | --- | --- |
| Test Name | Conc (µg mL^−1^) | Peak R_t_ | Peak Area | Conc (µg mL^−1^) | Peak R_t_ | Peak Area | Conc (µg mL^−1^) | Peak R_t_ | Peak Area | Conc (µg mL^−1^) | Peak R_t_ | Peak Area |
| Standard | 40.0 | 1.310 | 625.9 | 4.0 | 2.092 | 97.5 | 4.2 | 3.589 | 115.0 | 40.0 | 7.791 | 2474.4 |
| Test | 40.0 | 1.314 | 629.3 | 4.0 | 2.090 | 96.8 | 4.2 | 3.586 | 108.4 | 40.0 | 7.824 | 2480.1 |
| Placebo | 0 | No Peak | No Peak | 0 | No Peak | No Peak | 0 | No Peak | No Peak | 0 | No peak | No peak |
